# A Multifactorial Model to Predict the Surgical Complexity of Lung Resection After Neoadjuvant Chemoimmunotherapy

**DOI:** 10.1016/j.atssr.2025.09.014

**Published:** 2025-10-16

**Authors:** Alessandro Brunelli, Marco Nardini, Joshil Lodhia, Nilanjan Chaudhuri, Richard Milton, Elaine Teh, Peter Tcherveniakov, Kostas Papagiannopoulos, Katy Clarke, Pooja Bhatnagar, Kevin Franks

**Affiliations:** 1 Department of Thoracic Surgery, St James’s University Hospital, Leeds, United Kingdom; 2 Department of Oncology, St James’s University Hospital Leeds, United Kingdom

## Abstract

**Background:**

This study aims to develop an predictive model for surgical complexity after neoadjuvant chemoimmunotherapy.

**Methods:**

This is real clinical practice study of consecutive patients undergoing surgery after neoadjuvant nivolumab and chemotherapy for locally advanced lung cancer (April 23, 2023 through December 24, 2024). Surgical complexity was graded by the operating surgeon using a 4-dimension published score. An operation was defined as complex if at least 1 of the dimensions scored severely more complex than a standard lobectomy. Logistic regression analysis was used to test the association of several patient- and tumor-related factors with the presence of a complex procedure. The model was constructed by proportionally weighing the regression coefficients.

**Results:**

The analysis included 65 patients. The most frequent procedure was lobectomy (86%), of which 28 cases (43%) were classified as complex. Complex procedures were longer (median 228 minutes vs 180 minutes; *P* = .003) and were more frequently converted to thoracotomy (48% vs 6.7%, *P* = .001). Logistic regression analysis showed that absence of radiologic nodal response to neoadjuvant treatment (score, 1 point; regression coefficient, 2.4; *P* = .007), pretreatment cN2 stage (score 1 point; regression coefficient, 2.1; *P* = .024), and programmed death-ligand 1 ≥50% (score 1 point; regression coefficient, 1.67; *P* = .012) were independent predictors of a complex procedure. The final predictive surgical complexity model ranged from 0 to 3. The proportion of complex operations significantly increased with the higher risk scores (ie, 0% in patients with no risk factors, to 100% in those with all 3 risk factors, *P* < .001).

**Conclusions:**

The proposed model can assist to efficiently planning lung cancer surgery in the neoadjuvant setting and for patient counseling.

## Visual Abstract

**Figure:**
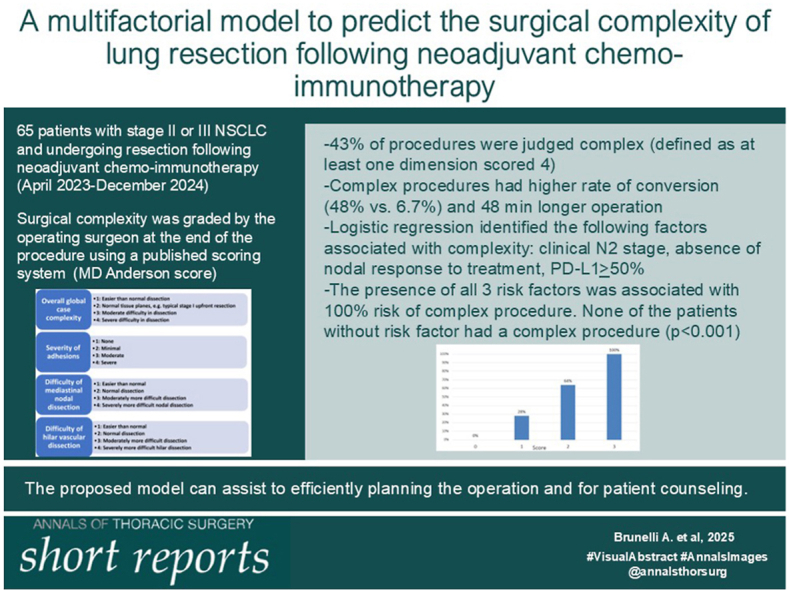

**In Short**

▪ Of lung resections after chemoimmunotherapy, 43% were judged severely more complex than a standard lobectomy.
▪ The absence of radiologic nodal response to neoadjuvant treatment, the presence of pretreatment cN2 stage, and programmed death-ligand 1 ≥50% were independent predictors of a complex procedure.
▪ The presence of all 3 factors was associated with a 100% risk of a complex procedure.

Recent studies suggest that 45% to 60% of lung resections after neoadjuvant chemoimmunotherapy are technically more demanding than standard lobectomy procedures.^1^^,^^2^ These challenges typically arise from fibrosis, adhesions, and difficult vascular or nodal dissections. Despite these reports, evidence remains limited regarding quantifying surgical complexity and identifying reliable preoperative predictors.

This study aimed to develop a predictive risk model for surgical complexity in patients undergoing lung resection after neoadjuvant chemoimmunotherapy. A practical tool for anticipating operative difficulty could support multidisciplinary planning, inform patient counseling, and promote safer delivery of care.

## Patients and Methods

### Study Design and Population

The study was approved by the Information Governance and Research and Innovation Departments at Leeds Teaching Hospitals (IRAS no. 345443, Ref. LTH24040; approval date, July 1, 2024). Owing to the retrospective design and use of anonymized data, individual patient consent was waived.

We analyzed consecutive patients undergoing anatomical lung resection between April 2023 and December 2024 after 3 cycles of neoadjuvant platinum-based chemotherapy combined with nivolumab for resectable stage II to IIIB non-small cell lung cancer (NSCLC). This was the only approved neoadjuvant immunotherapy regimen in England during the study period; therefore, all patients received this protocol.

Eligibility was determined through a multidisciplinary tumor board. Patients had histologically confirmed, resectable stage II to IIIB NSCLC (Eighth TNM edition), with no epidermal growth factor receptor or anaplastic lymphoma kinase alterations. All operations were performed by board-certified thoracic surgeons in a dedicated thoracic unit.

### Surgical Complexity Assessment

Surgical complexity was graded using the MD Anderson score,^3^ which incorporates overall operative difficulty, severity of pleural adhesions, and challenges in mediastinal or vascular dissection due to fibrosis (Supplemental Table S1). The score was applied to classify procedures as complex or noncomplex. This study focused on identifying preoperative factors predicting complexity rather than validating the scoring tool itself.

### Statistical Analysis

Details of the statistical methods are provided in the Supplemental Material. In brief, univariable and multivariable logistic regression were used to identify predictors of complexity. A stepwise elimination approach was applied to construct the predictive model.

## Results

### Patient and Surgical Characteristics

A total of 65 patients met inclusion criteria (Supplemental Table S2). A minimally invasive approach was initiated in 53 resections (82%): 46 video-assisted thoracoscopic (VATS) and 7 robotic. Conversion to thoracotomy occurred in 13 cases (24.5%), most often due to bleeding (n = 5), scarring around the pulmonary artery (n = 5), pleural adhesions (n = 2), or bulky fibrotic hilar nodes (n = 1).

Lobectomy was the most common operation (n = 56 [86%]), followed by bilobectomy (n = 5 [7.7%]) and pneumonectomy (n = 4 [6.2%]). As determined by the global score, 77% of procedures were more complex than a standard lobectomy (Supplemental Table S3), with 23 operations (43%) categorized as severely complex, defined by at least 1 score dimension rated as grade 4.

### Surgeon Experience

Consultants with <5 years of practice performed 46 procedures (71%). The proportion of complex operations did not differ significantly between junior and senior surgeons (43% vs 42%, *P* = 1.0).

### Operative Outcomes

The results of the comparison of outcomes between complex and noncomplex procedures are reported in Table 1. Complex procedures were associated with significantly longer operating times, greater blood loss, and higher conversion rates. Nearly half of complex resections required conversion compared with <10% of noncomplex cases. Operative approach (VATS vs robotic) did not influence complexity rate (41% vs 57%, *P* = .45) or conversion risk.

**Table 1:** Surgical Outcomes After Complex and Noncomplex Procedures

Variable	Complex	Noncomplex	*P* Value
	(n = 28)	(n = 37)	
Surgical time, min	228 (181-265)	180 (138-216)	.003
Blood loss, mL	200 (100-350)	100 (50-200)	.083
Conversions	11/23 (48)	2/30 (6.7)	.001
Length of stay, d	6 (4-8)	5 (4-8)	.63
Adverse event grade 3+	5 (17.6)	4 (10.8)	.48
Complete pathologic response	8 (28)	10 (27)	1
Major pathologic response	19 (67)	16 (43)	.078

Results are expressed as median (interquartile range) for numeric variables and as count (%) for categoric variables.

Conversion increased from 7.6% when operations were judged noncomplex to 30% when procedures were deemed moderately or severely more difficult. However, length of stay, major (Clavien-Dindo ≥3) complications, and 90-day mortality (n = 2, 1 in each group) did not differ between complex and noncomplex operations. Rates of complete pathologic response and major pathologic response were also comparable.

### Predictors of Complexity

Univariable analysis found no significant association between complexity and demographic factors, tumor size, histology, central location, positron emission tomography maximum standard uptake value, or interval from treatment to surgery (Supplemental Table S4). Specifically, delaying surgery >6 weeks after treatment completion was not linked with increased complexity (44% vs 43%, *P* = .89).

Clinical nodal stage showed a trend: cN2 disease accounted for 63% of complex cases compared with 25% of cN1 and 39% of cN0 (*P* = .07). Absence of radiologic nodal response was strongly associated with complexity (62% vs 29%, *P* = .03).

Programmed death-ligand 1 (PD-L1) expression was available for 57 patients. High PD-L1 (≥50%) was present in 49% and correlated with greater surgical difficulty (61% complex vs 28% in low PD-L1, *P* = .012).

### Multivariable Model

Stepwise logistic regression identified 3 independent predictors of surgical complexity:

• Pretreatment cN2 disease (odds ratio, 2.1; 95% CI, 0.3-3.9; *P* = .024)
• Absence of radiologic nodal response (odds ratio, 2.4; 95% CI, 0.7-4.1; *P* = .007)
• PD-L1 ≥50% (odds ratio, 1.7; 95% CI, 0.4–3.0; *P* = .012)

Each factor was assigned 1 point to create the Predictive Surgical Complexity Model (PSCM) ranging from 0 to 3.

The probability of complex surgery rose progressively with increasing PSCM score (Table 2, Figure). Notably, none of the patients with PSCM = 0 had blood loss >350 mL or operating time >240 minutes, whereas 30% to 37% of patients with PSCM ≥2 exceeded these thresholds. However, conversion rates did not rise significantly across PSCM categories (0%-32%, *P* = .62).

**Table 2:** Incidence of Complex Procedures by Class of Risk According to the Predictive Surgical Complexity Model

Predictive Surgical Complexity Score	Patients in Each Class of Risk	Patients With Complex Operations
	No. (%)	No. (%)
0	6 (9.2)	0
1	32 (49.2)	9 (28)
2	22 (33.9)	14 (64)
3	5 (7.7)	5 (100)

**Figure:**
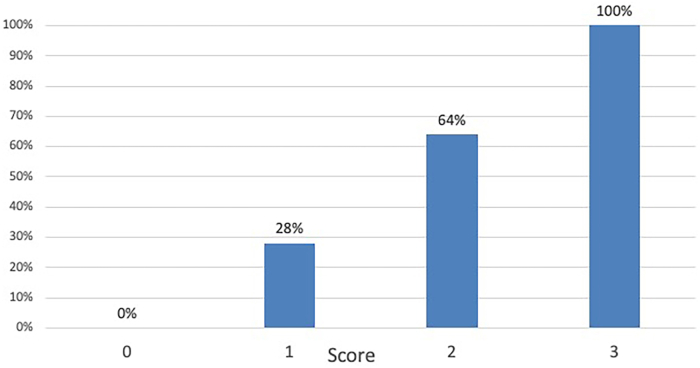
Distribution of complex procedures by predictive surgical complexity score.

## Comment

In this study, 43% of lung resections after neoadjuvant nivolumab immunotherapy were deemed severely complex, consistent with prior reports.^1^^,^^2^ Complex procedures were marked by longer duration, higher blood loss, and a 7-fold increase in conversion to thoracotomy. Importantly, pathologic response was not linked with operative complexity, echoing earlier findings.^1^^,^^2^

We identified 3 key predictors: pretreatment cN2 stage, lack of nodal radiologic response, and PD-L1 ≥50%. Each likely contributes through distinct mechanisms.

• cN2 stage reflects more locally advanced disease, increasing the technical difficulty of dissection.
• Absence of radiologic nodal response may represent persistent bulky or fibrotic nodes obstructing hilar access. Whereas some studies suggested that fibrosis after nodal regression complicates dissection,^2^ others found that residual disease was more strongly associated with difficult minimally invasive surgery and higher conversion risk.^4^, ^5^, ^6^ Our findings align with the latter.
• High PD-L1 expression may indicate an inflamed tumor microenvironment, with cytokine-driven fibrosis and adhesions.^7^ It is also a recognized marker of tumor aggressiveness, potentially correlating with anatomical features that complicate resection.^8^

The PSCM provided clear risk stratification. Patients with no risk factors had straightforward procedures, whereas those with all 3 invariably underwent complex resections. Although the model was not designed to predict hard outcomes, such as conversion or blood loss, it effectively anticipated technical difficulty, making it clinically useful for preoperative planning.

### Limitations

Several limitations must be acknowledged. This was a single-center study in a high-volume academic unit; findings may not generalize to different settings. Complexity was defined using a published but subjective scoring system. Operative judgment is inherently influenced by surgeon experience and potential confirmation bias. Additionally, most operations were performed with VATS; robotic resections were few, limiting conclusions about robotic strategies.

The study period coincided with the early adoption of nivolumab as a neoadjuvant agent. A potential learning curve effect cannot be excluded. Moreover, surgeon case mix in our unit may not mirror other centers. Independent external validation is required.

### Conclusions

We developed a simple risk score (PSCM) that stratifies the likelihood of complex lung resection after neoadjuvant chemoimmunotherapy. The model, based on pretreatment cN2 stage, lack of radiologic nodal response, and PD-L1 ≥50%, can inform operative planning, multidisciplinary discussions, and patient counselling.

At our institution, anticipated complex resections are now scheduled as dual-consultant operations, discussed at high-risk multidisciplinary tumor board meetings, and approached with a lower threshold for conversion. Although all procedures are still initiated minimally invasively, awareness of risk facilitates preparedness for intraoperative challenges.

Future work will focus on prospective validation in external cohorts and exploration of whether PSCM can be adapted to predict additional outcomes such as conversion or blood loss.
