# Supplementary material for: A Multifactorial Model to Predict the Surgical Complexity of Lung Resection After Neoadjuvant Chemoimmunotherapy
Source: Ann Thorac Surg Short Rep. 2025 Oct 16;4(1):224–8. doi: 10.1016/j.atssr.2025.09.014 (PMC13100776; doi:10.1016/j.atssr.2025.09.014)
Supplement: Supplementary Material [file mmc1.docx]

***Statistical analysis*:** For this study an operation was defined as complex if at least one of the four domains were graded as severely more complex than a standard lobectomy (4 points).

The following patient- and tumour-related factors were tested for an association with the presence of a complex procedure: age, sex, forced expiratory volume in one second (FEV1), carbon monoxide lung diffusion capacity (DLCO), presence of COPD, history of coronary artery disease, cerebrovascular disease, diabetes, PD-L1 expression level, location of tumour (central as opposed to peripheral), side of tumour, histology (adenocarcinoma vs non adenocarcinoma), size of tumour prior to systemic treatment, baseline PET SUV max of the primary tumour, clinical nodal stage, percentage response of primary tumour to systemic treatment, presence of radiologic response of nodal disease to SACT, delay to surgery(>6 weeks from last cycle). For this study, we used sex to define a set of biological attributes that are associated with physical and physiological features. A binary sex categorization (male/female) is usually designated at birth. For this analysis, absence of lymph node response was defined as an absence of downstaging of cN1 or cN2 disease or in those patients with cN0 the absence of upstaging (no changes from pre-treatment stage).

Univariable analysis was used to screen variables to be used in a stepwise logistic regression with back ward elimination of variables. The Shapiro Wilk test was used to assess normal distribution of numeric variables. Numeric variables with normal distribution were tested using an unpaired Student t test whiles those without normal distribution were tested using the Mann Whitney test. Chi-square or Fisher’s exact tests were used to analyse categorical variables.

Variables with a p<0.2 at univariable analysis and additional clinically meaningful factors (nodal radiologic response to SACT, clinical N stage, central location of tumour, size of tumour prior to SACT, PET SUVmax of primary tumour, PD-L1 expression) were initially entered in the logistic regression analysis. A p value <0.10 was used to retain variables in the model.

The predictive surgical complexity model (PSCM) was constructed by proportionally weighing the regression coefficients of the variables retained in the final model and assigning 1 point to the lowest one. Each patient was assigned a PSCM derived from the sum of the points if any. Patients were then grouped into classes of risk according to their PSCM and reflecting a similar incidence of complex operations. This methodology was previously used to construct similar aggregate scores (1)

All tests were performed using the Stata 15.0 statistical software (Stata Corp, College Station, TX, USA).

Reference:

1. Pompili C, Falcoz PE, Salati M, Szanto Z, Brunelli A. A risk score to predict the incidence of prolonged air leak after video-assisted thoracoscopic lobectomy: An analysis from the European Society of Thoracic Surgeons database. J Thorac Cardiovasc Surg. 2017 Apr;153(4):957-965

**Table S1:** Score used to grade complexity of lung resection following neoadjuvant chemo-immunotherapy

| Overall global case complexity | 1. Easier than normal dissection |
| --- | --- |
|  | 1. Normal tissue planes, e.g. typical stage I upfront resection |
|  | 1. Moderate difficulty in dissection |
|  | 4.Severe difficulty in dissection |
| Severity of adhesions | 1. None |
|  | 1. Minimal |
|  | 1. Moderate |
|  | 1. Severe |
| Difficulty of mediastinal nodal dissection | 1. Easier than normal |
|  | 1. Normal nodal dissection |
|  | 1. Moderately more difficult nodal dissection |
|  | 1. Severely more difficult nodal dissection |
| Difficulty of hilar vascular dissection | 1.Easier than normal hilar dissection |
|  | 2. Normal hilar vascular dissection |
|  | 3.Moderately more difficult hilar dissection |
|  | 4.Severely more difficult hilar dissection |

**Table S2:** Characteristics of the patients included in the analysis (no. 65)

| Age | 65.3 (10) |
| --- | --- |
| Sex males (n,%) | 38 (58%) |
| FEV1% | 88.6 (18.8) |
| DLCO% | 79.7 (15.6) |
| PS  0  1 | 38 (58.5%)  27 (41.5%) |
| CAD (n,%) | 5 (7.7%) |
| current smoker (n,%) | 26 (40%) |
| Histology  Adenocarcinoma  squamous cell | 37 (57%)  28 (43%) |
| PD-L1>1% (measured in 57 patients) | 40 (70%) |
| Clinical stage  IIA  IIB  IIIA  IIIB | 2 (3%)  23 (35%)  35 (54%)  5 (8%) |

Results are expressed as means and standard deviations or count and percentages for categorical variables. FEV1: forced expiratory volume in one second; DLCO: carbon monoxide lung diffusion capacity; PS: ECOG performance status; CAD: history of coronary artery disease; PD-L1: Programmed Cell Death Ligand 1

**Table S3:** Distribution of the complexity as judged by the operating surgeons at the end of the procedure

|  | **Easier than normal** | **Normal dissection (typical stage I upfront resection)** | **Moderate difficulty in dissection** | **Severe difficulty in dissection** |
| --- | --- | --- | --- | --- |
| Overall global complexity | 1 (1.5%) | 14 (21.5%) | 39 (60%) | 11 (17%) |
| Mediastinal nodal dissection | 3 (4.5%) | 22 (34%) | 26 (40%) | 14 (21.5%) |
| Hilar vascular dissection | 4 (6%) | 15 (23%) | 29 (45%) | 17 (26%) |
| Pleural adhesions | None 26 (40%) | Minimal 15 (23.1%) | Moderate 19 (29.2%) | Severe 5 (7.7%) |

**Table S4:** comparison of patient and tumour related characteristics between complex and non-complex operations

| Variables | Complex (n. 28) | non-complex (n. 37) | p-value |
| --- | --- | --- | --- |
| age | 66.7 (9.3) | 64.2 (10.4) | 0.31 |
| sex males | 18 (64%) | 20 (54%) | 0.41 |
| FEV1% | 86.1 (18.5) | 90.6 (19.0) | 0.39 |
| DLCO% | 79.3 (15.4) | 80.0 (15.9) | 0.75 |
| COPD | 9 (32%) | 10 (27%) | 0.78 |
| Location of the tumour (central) | 17 (61%) | 21 (57%) | 0.75 |
| side of the tumour (right) | 17 (68%) | 24 (65%) | 0.52 |
| Histology (adenocarcinoma) | 16 (57%) | 21 (57%) | 0.98 |
| size of the tumour before treatment (cm) | 5.3 (2.4) | 4.9 (2.9) | 0.38 |
| PET-SUVmax primary tumour before treatment | 16 (8.0) | 14.1 (7.6) | 0.32 |
| percentage response of primary tumour to systemic treatment | 31% (95% CI 19-43) | 32% (95% CI 21-43) | 0.85 |

Results are expressed as means and standard deviations or count and percentages for categorical variables. FEV1: forced expiratory volume in one second; DLCO: carbon monoxide lung diffusion capacity; COPD: moderate to severe Chronic Obstructive Pulmonary Disease
